# Pay-it-forward gonorrhea and chlamydia testing among men who have sex with men in China: a study protocol for a three-arm cluster randomized controlled trial

**DOI:** 10.1186/s40249-019-0581-1

**Published:** 2019-08-16

**Authors:** Tiange P. Zhang, Fan Yang, Weiming Tang, Marcus Alexander, Laura Forastiere, Navin Kumar, Katherine Li, Fei Zou, Ligang Yang, Guodong Mi, Yehua Wang, Wenting Huang, Amy Lee, Weizan Zhu, Peter Vickerman, Dan Wu, Bin Yang, Nicholas A. Christakis, Joseph D. Tucker

**Affiliations:** 1 University of North Carolina at Chapel Hill Project-China, No. 2 Lujing Road, Guangzhou, 510095 China; 2 Loyola University Chicago Stritch School of Medicine, Maywood, IL USA; 3 School of Medicine, University of North Carolina at Chapel Hill, Chapel Hill, North Carolina USA; 4 Southern Medical University Dermatology Hospital, Guangzhou, China; 5 Human Nature Lab, Department of Sociology, Yale University, New Haven, CT USA; 6 Weill Cornell Medical College, New York, NY USA; 7 Department of Biostatistics, University of North Carolina at Chapel Hill, Chapel Hill, North Carolina USA; 8 Blued, Beijing, China; 9 Zhitong Guangzhou LGBT Center, Guangzhou, China; 10 School of Social and Community Medicine, University of Bristol, Bristol, UK; 11 Faculty of Infectious and Tropical Diseases, London School of Hygiene and Tropical Medicine, London, UK

**Keywords:** Men who have sex with men, Gonorrhoea and chlamydia testing, Pay-it-forward, Integrated HIV testing, China, Randomized controlled trial

## Abstract

**Background:**

Gonorrhea and chlamydia testing rates are poor among Chinese men who have sex with men (MSM). A quasi-experimental study suggested that a pay-it-forward strategy increased dual gonorrhea/chlamydia testing among MSM. Pay-it-forward offers an individual a gift (e.g., a free test) and then asks the same person if they would like to give a gift to another person. This article reports the protocol of a randomized controlled trial to evaluate dual gonorrhea/chlamydia test uptake and other outcomes among MSM in three arms – a pay-it-forward arm, a pay-what-you-want arm, and a standard of care arm.

**Methods:**

Three hundred MSM will be recruited at three HIV testing sites in Guangzhou and Beijing. Testing sites include two hospital-based MSM sexually transmitted diseases clinics and one MSM community-based organization. Eligible participants will be born biologically male, aged 16 years or older, reporting previous anal sex with another man, having never participated in the pay-it-forward program, without previous gonorrhea and chlamydia testing in the past 12 months, and residing in China. Following a cluster randomized design, every cluster of ten participants will be randomly allocated into one of three arms: (1) a pay-it-forward arm in which men are offered free gonorrhea and chlamydia testing and then asked whether they would like to donate (“pay it forward”) toward testing for future testers; (2) a pay-what-you-want arm in which men are offered free testing and told to decide how much to pay after receiving the test; (3) a standard of care arm in which men can pay the full price for dual gonorrhoea and chlamydia testing. The primary outcome is dual gonorrhoea/chlamydia testing as verified by administrative records. Secondary outcomes include incremental cost per test, incremental cost per diagnosis, community connectedness, and social cohesion. Primary outcome will be calculated for each arm using intention-to-treat and compared using one-sided 95% confidence intervals with a margin of 20% increase defined as superiority.

**Discussion:**

This study will examine the pay-it-forward strategy in comparison to the standard of care in improving test uptake for gonorrhea and chlamydia. We will leverage the cluster randomized controlled trial to provide scientific evidence on the potential effect of pay-it-forward. Findings from this study will shed light on novel intervention methods for increasing preventive health service utilization and innovate ways to finance it among communities.

**Trial registration:**

ClinicalTrials.gov, NCT03741725. Registered on 12 November 2018.

**Electronic supplementary material:**

The online version of this article (10.1186/s40249-019-0581-1) contains supplementary material, which is available to authorized users.

## Multilingual abstracts

Please see Additional file 1 for translations of the abstract into the five official working languages of the United Nations.

## Background

Gonorrhea and chlamydia are among the most prevalent sexually transmitted infections (STIs) in China [1, 2]. Men who have sex with men (MSM) is a recognized high risk group for STIs and is estimated to have a population of 2–10 million in China [1, 3]. One study conducted from 2015 to 2017 among MSM in Guangzhou reported an overall gonorrhea prevalence of 12.5% and a chlamydia prevalence of 18.1% [2]. Gonorrhea and chlamydia infections are also associated with increased risk for HIV transmission and acquisition [4, 5]. Yet, infections are commonly asymptomatic and easily missed without screening, especially those at extragenital sites [6–8]. For MSM, the World Health Organization recommends periodic testing for asymptomatic urethral and rectal gonorrhea and chlamydia using nucleic acid amplification tests (NAAT) [9]. US Centers for Disease Control and Prevention (CDC) also suggests annual NAAT testing for gonorrhea and chlamydia in asymptomatic MSM who are at high risk [10].

Yet, gonorrhea and chlamydia testing rate remains low among MSM in China [11], where studies show that less than 50% of MSM have ever been tested [12, 13]. Factors that contribute to poor gonorrhea and chlamydia testing among Chinese MSM include poor government funding for testing programs [14], inadequate linking of STI testing to existing HIV testing systems [15, 16], and insufficient MSM community involvement in STI efforts other than HIV. First, few government incentives exist to support the additional training and laboratory technology associated with regular gonorrhea and chlamydia testing [14]. Second, while many HIV testing programs for key populations have began to offer syphilis testing, most are still not set up to facilitate comprehensive STI testing [15, 16]. Third, MSM community organizations have had an increasing yet still limited role in China’s HIV/STI response [17–19]. Most community-based outreach programs prioritize HIV, with few focused on gonorrhea or chlamydia [20].

In light of this need, several community organizations collaborated with public health staff to organize a gonorrhea and chlamydia testing pilot program using a pay-it-forward strategy [21]. In pay-it-forward, one person is offered a gift (e.g., a free test), then they are asked whether they would like to give a gift to another person [22, 23]. Pay-it-forward has been studied in behavioral economics as a form of consumer elective pricing, defined as an economic transaction that allows individuals to purchase goods or services for any price (including zero) [24]. Pay-it-forward chains of giving is a type of cooperative behavior that can take place between known or anonymous individuals [25]. In social science experiments, pay-it-forward has been shown to promote generosity and engage group cohesiveness [22, 24].

One pilot study found that pay-it-forward increased gonorrhea/chlamydia testing 19-fold, with gonorrhea/chlamydia testing received by 53.7% (109/203) in the pay-it-forward group and 5.9% (12/205) in the standard of care group [21]. However, this was not a randomized controlled trial (RCT) and the study only compared two groups of individuals. We will evaluate the pay-it-forward model using a three-arm cluster randomized controlled trial. The purpose of this trial is to evaluate a pay-it-forward intervention for increasing gonorrhea and chlamydia testing among Chinese MSM.

## Methods/design

### Specific aims

1. Aim 1: To compare the effectiveness of a pay-it-forward program to standard of care in promoting gonorrhea/chlamydia testing among Chinese MSM. Hypothesis: Pay-it-forward is superior to the standard of care in promoting gonorrhea/chlamydia testing.
2. Aim 2: To compare the effectiveness of a pay-what-you-want program to standard of care in promoting gonorrhea/chlamydia testing among Chinese MSM. Hypothesis: Pay-what-you-want is superior to the standard of care in promoting gonorrhea/chlamydia testing.
3. Aim 3: To determine the gonorrhea/chlamydia cost per test and the cost per case diagnosed within the three trial arms. Hypothesis: Pay-it-forward is associated with lower cost per test and lower cost per case diagnosed.

### Methods

#### Trial design and timeline

This study will be a three-arm cluster randomized controlled superiority trial, with the main comparisons being pay-it-forward vs standard of care and pay-what-you-want vs standard of care. Men will be randomly assigned in a 1:1:1 ratio into three arms. The three arms of the study will include: (1) a pay-it-forward arm in which men are offered free gonorrhea and chlamydia testing and then given the option to donate (“pay it forward”) toward testing for future testers; (2) a pay-what-you-want arm in which men are offered free testing and told to decide how much to pay after receiving the test; and (3) a standard of care arm in which men can pay the full price for dual gonorrhoea and chlamydia testing (RMB 150, or USD 22). Men will be recruited at three sites: two hospital-based MSM sexually transmitted disease (STD) clinics in Guangzhou and one MSM community-based organization in Beijing.

Following a cluster randomized design, every cluster of ten participants in each of the three study sites will be randomly allocated into one of the three arms independently: pay-it-forward arm, pay-what-you-want arm, or the standard of care arm. The rationale for choosing ten participants per cluster as the unit of randomization rather than individual participants is to minimize contamination of intervention across individuals and to facilitate cooperation with clinic staff. Gonorrhoea/chlamydia testing is performed immediately following each trial arm. Gonorrhoea/chlamydia test uptake will be ascertained by assessing administrative records. Eligible men will also complete a single survey (see Additional file 2). No follow-up surveys will be conducted in this study.

This study will span approximately 3 months. The first month will consist of RCT preparations and a pilot for the RCT. The following period will consist of RCT recruitment and implementation. Recruitment will continue at all sites following a cluster randomization process until the pre-determined sample size is reached - a process that is expected to take approximately 1.5 months. The study will be managed by the Social Entrepreneurship to Spur Health (SESH) group, a multi-sectoral implementation research collaboration led by the Southern Medical University Dermatology Hospital and the University of North Carolina Project-China.

#### Study setting and recruitment

Guangzhou and Beijing are large metropolitan areas with high burdens of STDs and HIV among MSM. The RCT will enroll from an STD clinic and a local MSM community-based organization (CBO) sites that provide free HIV testing for MSM. The STD clinic site operates within an ongoing hospital-based program; the CBO testing site operates within an ongoing community-based program. All sites already provide free HIV testing to MSM, and are staffed by MSM volunteers, nurses, and public health staff. There are no clinical doctors at either site. The staff at each site is responsible for handling blood draws, testing, results reporting, and follow-up of HIV tests. All sites will follow the same study procedures.

#### Eligibility criteria

Potential participants must first agree to a print informed consent in order to participate. Inclusion criteria are: born biologically male, age 16 years or older, report previous anal sex with another man, never participated in the pay-it-forward program, and report one of the following with regards to gonorrhoea/chlamydia testing history: not tested for both gonorrhoea and chlamydia in the past 12 months, report testing for both gonorrhoea and chlamydia in the past 12 months with high-risk sexual behaviour following testing, report previous testing for either or both gonorrhoea and chlamydia but does not recall whether tested within the past 12 months. All eligible participants must provide a working unique mobile phone number or WeChat account identification to be enrolled. These will be used only for results notification.

#### Interventions

##### Pay-it-forward arm

**Fig. 1:**
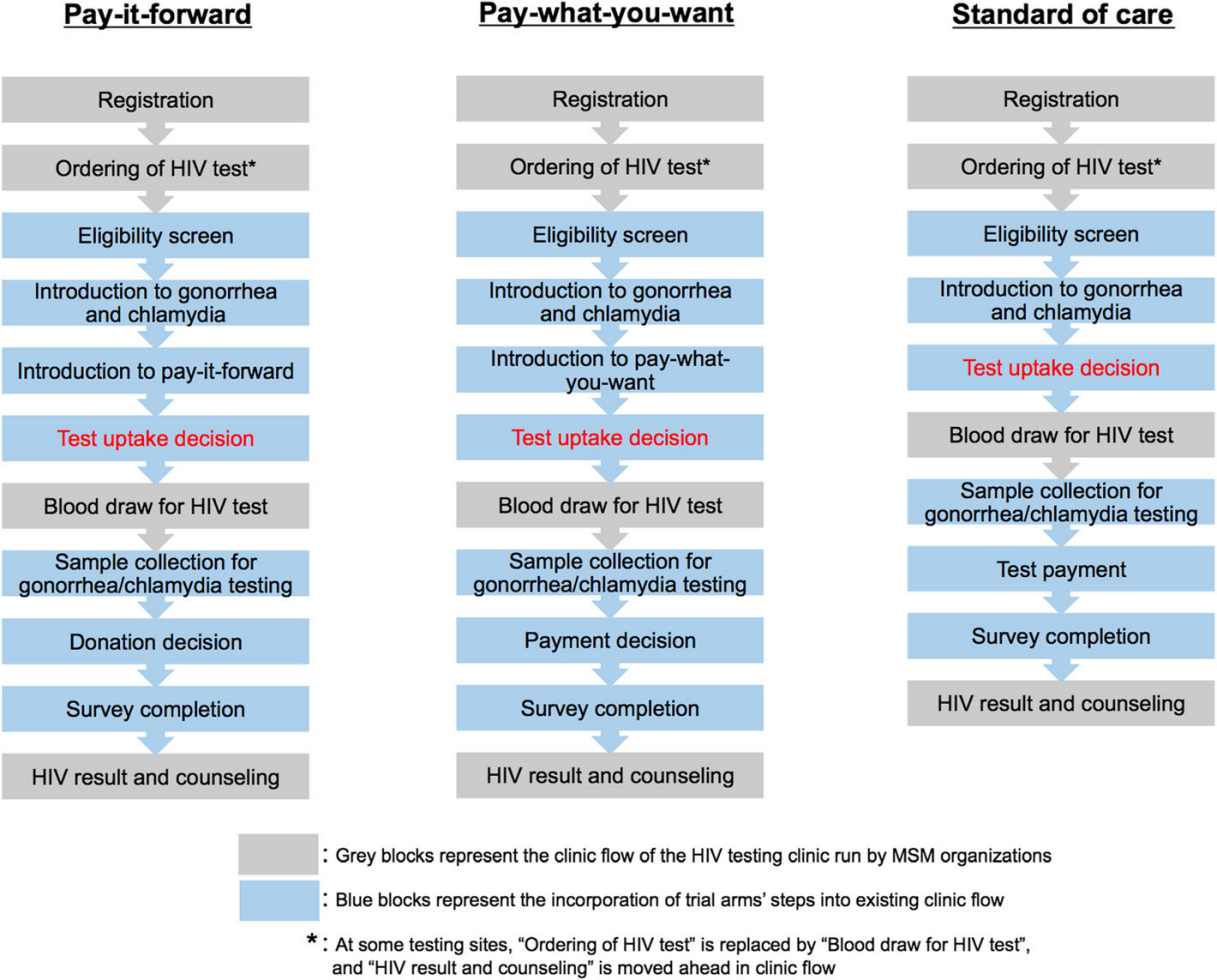
Schematic of three trial arms. The block diagrams describe the implementation steps for each arm

The pay-it-forward (PIF) program is developed using crowdsourcing. An open challenge contest [26] developed the name and format of the program materials. Hand-written postcards from previous participants in the program will encourage testing among future participants. Community volunteers will provide inputs to the program. At all program sites, MSM will be offered dual gonorrhea/chlamydia testing during their appointments for HIV testing (Fig. 1). First, all men will be provided a brief (5 min) introduction to gonorrhea and chlamydia testing using a pamphlet (see Additional file 3). Program organizers will then explain the pay-it-forward program to each man using a pamphlet (see Additional file 4). Briefly, men will be told that the hospital list price of gonorrhea and chlamydia testing is RMB 150 (USD 22), and that previous men attending the clinic had donated money to cover the cost of this test. Thus, each man could receive gonorrhea and chlamydia testing for free, then decide whether to donate money (pay-it-forward) for future men to have the same option. Men will be assured that donating is voluntary and told to pay any amount as they desire. A combination of initial research funding and donations from previous participants will be used to cover each man’s gonorrhea and chlamydia testing fees. Men will also be shown a postcard with a message written in simplified Chinese characters by a previous pay-it-forward contributor and told that they could also write a postcard message for a future participant. Men will then decide whether or not to receive combined gonorrhea and chlamydia testing. Regardless of their decision to test, men will be asked to fill out a brief survey about their sexual history, testing history, attitudes toward the testing program, and psychosocial conditions.

MSM who decide to test will be asked about their sexual practices and will be advised to consider receiving urine, anal, or both urine and anal dual gonorrhea/chlamydia testing. All participants will be advised that their information will be kept confidential, and that the results will be sent to them after approximately 1 week via WeChat, a phone- and computer-based instant messaging application. Men who agree to participate will choose to collect the sample on their own or have a staff member assist with sample collection [27]. After sample collection, men will decide whether or not to donate money to the next group of participants. Men can choose to leave donations for future participants with the program organizers via either cash or WeChat’s monetary transfer functionality. Each man will then be invited to write a message on a postcard for future pay-it-forward participants.

Program organizers will inform participants of their test results via WeChat. Samples collected in Guangzhou will be stored at room temperature overnight and then transported to Southern Medical University Dermatology Hospital in Guangzhou for laboratory testing within 1 week. Samples collected in Beijing will be shipped in insulated shipping container to Southern Medical University Dermatology Hospital for laboratory testing within 1 week. Patients who test positive will be counseled and directed to the WeChat page of the designated partnering hospital in each city, where they are able to make an appointment to receive treatment and follow-up.

##### Pay-what-you-want arm

Pay-what-you-want (PWYW) is a well-documented pricing strategy where the buyers decide and pay their desired amount for a given product or service, which is usually consumed by themselves [24]. Financially, pay-what-you-want is identical to pay-it-forward pricing: the customers choose the price they want to pay for a certain good or service, and the seller receives the payments. Socially, pay-what-you-want is different in that the customer is paying for him- or herself, while in pay-it-forward the customer is paying on behalf of someone else. Just as the pay-it-forward arm, MSM will be offered dual gonorrhea/chlamydia testing during their appointments for HIV testing. First, all men will be provided a brief (5 min) introduction to gonorrhea and chlamydia testing using a pamphlet. Program organizer will then explain the pay-what-you-want program to each man separately using verbal descriptions. Briefly, men will be told that the hospital list price of gonorrhea and chlamydia testing was RMB 150 (USD 22). Then they will be told that if they were to test today, they can participate in the pay-what-you-want program, in which they receive the test first and then decide how much they would like to pay. Men will be assured that payment is completely optional and advised to pay any amount that is desired by them. Men will then decide whether or not to receive urine, anal, or both urine and anal dual gonorrhea/chlamydia testing. Each man’s gonorrhea and chlamydia test fees will be covered by a combination of the initial research funding from the program organizers and payments from participants.

All participants will be ensured that their information will be kept confidential. Procedures for sample collection, sample processing, payment method, results reporting, and treatment linkage will be identical to those of the pay-it-forward arm.

##### Standard of care arm

Among men assigned to the standard of care arm, gonorrhea and chlamydia testing will be offered at the usual hospital list price (RMB 150, USD 22). All men will be provided a brief introduction about gonorrhea and chlamydia testing using the same pamphlet, but will not be introduced to other content of the above arms. Procedures for sample collection, sample processing, payment method, results reporting, and treatment linkage will be identical to those of the pay-it-forward arm.

#### Randomization and allocation

**Fig. 2:**
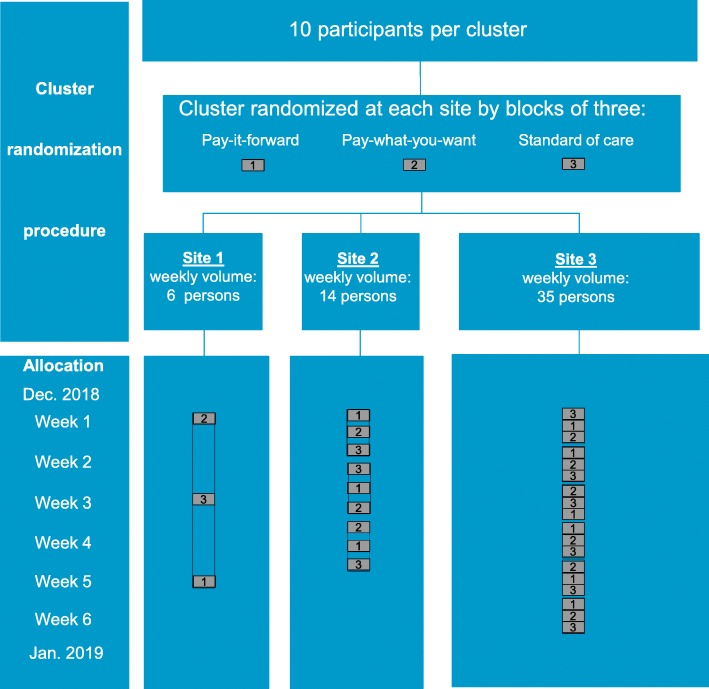
Schematic of cluster randomization procedure and sample allocation. This schematic diagram illustrates the cluster randomization procedure, average volume of testers who visit the study site for HIV tests during a typical week, and allocation. There are 30 clusters and 300 total participants, which satisfy the sample size. The sequence shown is for illustration and is not the actual allocation sequence

At each site, men will be assigned to one of the three arms through a cluster randomization procedure with ten participants per cluster (Fig. 2). To ensure equal number of clusters in each of the three arms, the clusters will be randomized in a block of three at each study site. The schedule will be determined prior to start of the study and then provided to each of the sites. STATA 15.1 software (StataCorp, StataCorp LLC, College Station, Texas) will be used to create the allocation sequence [28]. Ten blocks will be generated in total. Each block has three clusters consisting of pay-it-forward, pay-what-you-want, and standard of care, randomly created from all possible permutations.

Each eligible individual who presents to the site during a given 10-person cluster will be provided the allocated program. This RCT is not blinded. Participants will be able to determine their assignment based on whether they received intervention materials during the study period. Study organizers will be aware of randomization assignment because intervention materials provided to participants.

#### Laboratory testing

All urine and anal samples will be analyzed using Cobas 4800 CT/NG DNA detection kits (Roche Diagnostics, Basel, Switzerland) at Southern Medical University Dermatology Hospital. At the two MSM STD clinic sites, HIV testing will be performed using the Diagnostic Kit for Antibody to HIV ½ (Abon Pharmaceuticals, Northvale, New Jersey, USA) and syphilis testing, for those that choose to test, will be performed using the Treponema Pallidum Antibodies Rapid Test (Abon Pharmaceuticals, Northvale, New Jersey, USA). At the CBO site, HIV testing will be performed using the Third Generation Diagnostic Kit for Antibody to HIV rapid test (InTec, Xiamen, China) and syphilis testing, for those choosing to test, will be performed using the Syphilis Antibody (anti-Treponema Pallidum) rapid test (InTec, Xiamen, China).

#### Sample size

Sample size calculation (Table 1) is informed by a quasi-experimental study conducted in 2017–2018 at two similar study sites [21] and by a pilot study for the RCT conducted from October to November 2018. In the quasi-experimental study, gonorrhea/chlamydia testing was 53.7% in the pay-it-forward group and 5.9% in the standard of care group. In the pilot study for the RCT (Table 2), gonorrhea/chlamydia testing was 88% in the pay-it-forward group and 42% in the pay-what-you-want group. Based on these results and pilot data for the RCT, we assume that in the RCT we will have 54% test uptake in the pay-it-forward group, 42% test uptake in the pay-what-you-want group, and 6% in the standard of care. For the pay-it-forward uptake estimate, we chose to use the quasi-experimental study results (54%) instead of the pilot results (88%) because the quasi-experimental study was considered more similar in nature to our RCT, given that the pilot focused primarily on implementation optimization.

**Table 1:** Sample size calculations

Pay-it-forward and standard of care	ICC	0.01	0.05
	Proportion in control group	0.06	0.06
	Superiority margin	0.2	0.2
	Proportion in intervention group assuming H0	0.26	0.26
	Proportion in intervention group assuming H1	0.54	0.54
	Difference at which power is calculated	0.48	0.48
	Number of people per cluster	10	10
	Alpha	0.05	0.05
	Power	0.8	0.8
	Total sample size per arm	30	40
	Number of clusters per arm	3	4
Pay-what-you-want and standard of care	ICC	0.01	0.05
	Proportion in control group	0.06	0.06
	Superiority margin	0.2	0.2
	Proportion in intervention group assuming H0	0.26	0.26
	Proportion in intervention group assuming H1	0.42	0.42
	Difference at which power is calculated	0.36	0.36
	Number of people per cluster	10	10
	Alpha	0.05	0.05
	Power	0.8	0.8
	Total sample size per arm	80	110
	Number of clusters per arm	8	11

*ICC* Intra-class correlation

**Table 2:** Two piloted arms and the historical standard of care

Arms	Standard of care^a^	Pay-it-forward	Pay-what-you-want
Days piloted	52	4	5
Number of MSM offered intervention	205	17	26
GC/CT test uptake	12 (6%)	15 (88%)	11 (42%)
Number of MSM who contributed any amount	NA	15 (100%)	10 (91%)
Total contribution amount	NA	RMB 833.88 (USD 125.08)	RMB 465.99 (USD 69.90)
Average contribution Amount per tester	NA	RMB 55.59 (USD 8.34)	RMB 42.36 (USD 6.35)
Gonorrhea positive	NA	0	0
Chlamydia positive	NA	2	1

*MSM* Men who have sex with men

*GC/CT* Gonorrhea and chlamydia

*NA* Not applicable

^a^Historical standard of care data from a quasi-experimental study between March 11th and May 1st, 2018

We will have three arms -- pay-it-forward, pay-what-you-want, standard of care, with numbers of clusters at a ratio of 1:1:1. The trial between pay-it-forward and standard of care will be testing superiority in promoting gonorrhea/chlamydia test uptake at a superiority margin of 0.2 (20% difference in percentage agreeing to test in those two arms). This margin was chosen as a clinically significant difference in test uptake based on results from a modeling study [29]. Similarly, the trial between pay-what-you-want and standard of care will be also testing superiority with the same margin of 0.2. Assuming we will have three equal sized arms, with cluster size of 10 per cluster, alpha of 0.05, power of 0.8, inter class correlation of 0.01 or 005, we will at most need eight or 11 clusters per arm, respectively (Table 1). To integrate the sample size calculations and to consider practicality, the final sample size would be ten clusters in pay-it-forward, ten clusters in pay-what-you-want, and ten clusters in standard of care, with a total cluster size of 30 (300 participants).

#### Quality control

We will examine data collected from all participants. We will deduplicate participants by examining cell phone number and WeChat handle. The primary outcome for this study is uptake of dual gonorrhea/chlamydia testing by administrative record as a single outcome. For the pay-it-forward arm, we will record the amount that each participant donates to future participants. For the pay-what-you-want arm, we will record the amount that participants pay for their own test.

Information on socio-demographics, sexual behaviors, HIV/STI testing history, and psychosocial conditions will be collected using a standardized survey instrument. Socio-demographic characteristics include participants’ age, ethnicity, marital status, highest level of education completed, place of residency (urban or rural), and annual income level. Sexual behaviors and HIV/STI testing history include previous anal sex with men, role during anal sex, number of sex partners, condom use, sexual orientation disclosure, endorsement of STI symptoms, and testing history for HIV, gonorrhea, and chlamydia. Psychosocial variables include adapted measures for community engagement [30], community connectedness [31], identify fusion [32], and social cohesion [33, 34]. For all participants, we will collect HIV test results, as well as gonorrhea and chlamydia results for those who tested. We will also collect information on reasons for accepting or declining gonorrhea and chlamydia tests. For men assigned to the pay-it-forward group, we will also ask about perceived benefits and barriers of the pay-it-forward program. Upon completion of the study survey, participants will receive a pen valued approximately one USD.

#### Outcomes

The main outcome of this study will be gonorrhea and chlamydia testing by PCR as assessed by administrative records. The choice of diagnostics is based on World Health Organization and US CDC guidelines recommending regular gonorrhea/chlamydia testing among MSM with risks [9, 10]. Secondary outcomes will include incremental cost per test, incremental cost per diagnosis, community connectedness, and social cohesion. Effect modification analysis will include community engagement. Existing literature on community engagement [30], community connectedness [31, 32], and social cohesion [33, 34], informed the development of adapted scales in this study. Community engagement is measured using an adapted six-item yes/no scale used in a previous study on Chinese MSM [30]. A six-item community connectedness scale [31] and a seven-item social cohesion scale [33, 34] ask participants to rank their level of disagreement and agreement with each item using answer choices “Strongly Agree,” “Agree,” “Disagree” and “Strongly Disagree”. All three community scales were adapted to the local context and piloted among 43 Chinese MSM before RCT recruitment. Survey items are detailed in Additional file 2.

#### Confidentiality

Survey data will be collected partially from online-based questionnaire and then stored on a secure online survey platform (Wenjuanxing), and partially from paper-based questionnaire. Electronic data will be transmitted using 128-bit encryption across the Internet and mobile data networks. Paper-based data will be entered by research staff members on the same day as data collection. Responses to surveys, including participant mobile phone numbers and WeChat account numbers, will be stored on a secure server (electronic) and in a locked cabinet (offline), which can be accessed with login information known only to the research team.

#### Monitoring

A data monitoring committee will not be formed for this RCT because potential for harm to participants is minimal. If any person feels they have experienced an adverse event or unwanted effect from participating in this RCT, they can withdraw at any time. A telephone number and WeChat account will be provided to participants to contact the primary investigator with questions or concerns.

### Data analysis

#### Primary analysis

Socio-demographic characteristics will be summarized using descriptive statistics. The gonorrhoea/chlamydia test uptake rate will be calculated for each of the three arms. Generalized estimating equations will be used to account for potential correlation in outcomes due to clustering. The primary analysis will evaluate the hypotheses that the pay-it-forward intervention is superior to standard of care and that the pay-what-you-want intervention is superior to standard of care. The trial between pay-it-forward and standard of care will be testing superiority in promoting test uptake at a superiority margin of 20% in percentage agreeing to test in those two arms [29]. The trial between pay-what-you-want and standard of care will be testing with the same 20% margin. One-sided 95% confidence interval will be computed for pay-it-forward vs standard of care and for pay-what-you-want vs standard of care, and then compared to the superiority margin.

#### Subgroup analysis

Effect modification analyses will evaluate whether the effect of the pay-it-forward intervention on gonorrhoea/chlamydia test uptake varied in relation to the following factors: (1) younger age (less than 28 years old, 28 or older); (2) community-level constructs: community engagement (high or low level), community connectedness (high or low level), social cohesion (high or low level). For each factor of interest, three measures of association will be calculated: A) a crude measure of association between treatment group and test uptake, B1) a measure of association between treatment group and test uptake among all participants who report the factor of interest (younger age, or high level of a community-level construct), B2) a measure of association between treatment group and test uptake among all participants who deny a history of having the factor of interest (younger age, or high level of a community-level construct). Effect modification will be deemed present when B1 and B2 are different from one another, and at least one (B1 or B2) is different from A.

#### Missing data plan

Men will only be involved at a single time point and so there is no loss to follow up. However, there may be missing data in the primary and secondary outcomes. If the primary outcome is missing for < 15% of participants, analyses will use a complete-case approach. If an outcome is missing for ≥15% of participants, missingness mechanism will be investigated and multiple imputation will be used if suitable.

#### Secondary analyses

Within each arm of the study, we will calculate the incremental cost per test and the incremental cost per diagnosis. Cost will be collected from the perspective of the program provider. To estimate the program cost, we will identify the cost of each program element, such as personnel, facilities, equipment, and materials, and categorize costs into start-up costs, fixed costs, and variable costs. Start-up costs are incurred in the period between the decision to implement this trial and the start of trial recruitment (e.g. personnel training, testing sites coordination, pilot study). Fixed costs remain constant during the trial period regardless of the number of people served (e.g. administration, durable goods, and equipment). Variable costs are those that vary with the number of participants served (e.g. recruitment, counselling and testing, and nondurable goods and supplies). Personnel costs will be calculated by multiplying the staff time associated with each program activity by the compensation received by the staff who perform these activities. We will first calculate the total cost for each group, then divide these costs by the number of men tested and by the number of cases diagnosed with gonorrhea or chlamydia.

#### Pilot study results summary

To inform the development of this randomized controlled trial, the pay-it-forward and pay-what-you-want arms were evaluated through a pilot study conducted from October 2018 to November 2018 (Table 2).

A total of 43 eligible MSM were recruited at the selected study sites for the RCT. Recruitment took place on 9 days interspersed between 13 October and 15 November All men on a given day were assigned to either pay-it-forward or pay-what-you-want arm. In total, 17 men were recruited for the pay-it-forward arm over 4 days; 26 men were recruited for the pay-what-you-want arm over 5 days. Of these, 88% (15/17) of men received gonorrhea/chlamydia testing in the pay-it-forward arm and 42% (11/26) of men received gonorrhea/chlamydia testing in the pay-what-you-want arm. All men (100%, 15/15) in the pay-it-forward arm donated some amount, with average donation per participant of RMB 56. 91% (10/11) of men in the pay-what-you-want paid some amount, with average payment per participant of RMB 42. None were diagnosed with gonorrhea and three men were diagnosed with chlamydia.

These pilot results, along with results of a quasi-experimental study among 408 men [21], were used as the basis to inform sample size calculation. Through this pilot period, implementation steps and survey instrument were also optimized through an iterative process consisting of feedback from MSM participants, organizers, and community partners.

## Discussion

This trial will assess the effectiveness of pay-it-forward intervention in promoting gonorrhoea and chlamydia testing among Chinese MSM, a marginalized population where sustainable strategies for integrated HIV/STD testing programs are urgently needed. Few studies have evaluated the use of pay-it-forward interventions focused on improving health, and none to our knowledge have used a RCT design. This three-arm cluster RCT will demonstrate pay-it-forward’s ability to be effective in the complex context of local settings. The pay-it-forward model includes both free or subsidized testing and a sense of social obligation to contribute to the health of other gay men. With comparison to the pay-what-you-want arm, a model that is financially similar to pay-it-forward but socially contrasting, there is an opportunity to explore the extent to which social and financial factors contribute to pay-it-forward health programs.

Although this trial will yield important insights for STD testing programming in China, some limitations exist. The RCT will be conducted at sites of HIV/syphilis testing for MSM. This limits our sample to MSM who are undergoing HIV/syphilis testing and are connected with community-based organizations. The level of trust that MSM hold toward sites in which pay-it-forward programs are embedded may influence program effectiveness. The testing sites in this study are long-standing programs and trusted by the MSM community. Findings may be limited in generalizability to settings that lack existing HIV testing services or trusted sites for MSM.

Our study will generate important research and policy implications regarding the use of pay-it-forward to preventative health services. The study outcomes will help guide policy and intervention practice of governmental departments and community-based organizations regarding the expansion of key population STD testing strategies. Moreover, practical knowledge gained from developing and implementing an innovative, pay-it-forward intervention may be applicable for future efforts to build self-sustaining STD testing programs.

### Trial status

At the time of writing this article, RCT recruitment is ongoing. Data analysis for the RCT has not begun. The study is registered in the ClinicalTrials.gov database (NCT03741725). The database will also be used for documenting protocol modifications. The trial protocol conforms to the Standard Protocol Items: Recommendation for Interventional Trials (SPIRIT) 2013 statement.

## Additional files

Additional file 1: Multilingual abstracts in the five official working languages of the United Nations. (PDF 541 kb)

Additional file 2: Survey instrument (English version). This is the English version of the survey for our study. (DOCX 74 kb)

Additional file 3: Introduction to Gonorrhea and Chlamydia pamphlet (English version). This is the English version of the pamphlet used by study organizers to introduce Gonorrhea and Chlamydia to eligible men. (PPTX 779 kb)

Additional file 4: Introduction to Pay-It-Forward pamphlet (English version). This is the English version of the pamphlet used by study organizers to introduce pay-it-forward to eligible men. (PPTX 311 kb)

Additional file 5: Informed consent form (English version). This is the English version of the informed consent form for our study. (DOCX 47 kb)

## Data Availability

Not applicable.

## Abbreviations

CBO: Community-based organization
CDC: US Center for Disease Control and Prevention
H0: Null hypothesis
H1: Alternative hypothesis
LMIC: Low- and middle-income countries
MSM: Men who have sex with men
RCT: Randomized controlled trial
SESH: Social Entrepreneurship to Spur Health Group
