# Supplementary material for: Pay-it-forward gonorrhea and chlamydia testing among men who have sex with men in China: a study protocol for a three-arm cluster randomized controlled trial
Source: Infect Dis Poverty. 2019 Aug 16;8:76. doi: 10.1186/s40249-019-0581-1 (PMC6700988; doi:10.1186/s40249-019-0581-1)
Supplement: Supplementary file 1 — Multilingual abstracts in the five official working languages of the United Nations. (PDF 541 kb) [file 40249_2019_581_MOESM1_ESM.pdf]

Translation of the abstract into the five official working languages of the United Nations

اختبار السيلان والكلاميديا المدفوع للأمام بين الرجال الذين يمارسون الجنس مع الرجال في الصين: بروتوكول دراسة لمجموعة من ثلاثة ذراعين تجربة عشوائية محكومة

Tiange P. Zhang, Fan Yang, Weiming Tang, Marcus Alexander, Laura Forastiere, Navin Kumar, Katherine Li, Fei Zou, Ligang Yang, Guodong Mi, Yehua Wang, Wenting Huang, Amy Lee, Weizan uckerVickerman, Dan Wu, Bin Yang, Nicholas A. Christakis, Joseph D. T Zhu, Peter

نبذة مختصرة
اختبار السيلان والكلاميديا المدفوع للأمام بين الرجال الذين يمارسون الجنس مع الرجال في الصين: بروتوكول دراسة لمجموعة من ثلاثة ذراعين تجربة عشوائية محكومة اقترحت دراسة شبه تجريبية أن استراتيجية الدفع إلى الأمام زادت من اختبار السيلان / الكلاميديا المزدوج بين الرجال الذين يمارسون الجنس مع الرجال. يقدم forward-it-Pay للفرد هدية (على سبيل المثال ، اختبارًا مجانيًا) ثم يسأل نفس الشخص عما إذا كان يرغب في تقديم هدية لشخص آخر. توضح هذه المقالة بروتوكول تجربة معشاة ذات شواهد لتقييم الامتصاص المزدوج لاختبار السيلان / الكلاميديا والنتائج الأخرى بين الرجال الذين يمارسون الجنس مع الرجال في ثلاثة أذرع - ذراع الدفع إلى الأمام ، وذراع الدفع مقابل ما تريد ، ومستوى الرعاية ذراع.
الأساليب: ثلاث مقاطعات عينة في كل من المحافظات الثلاث: تم اختيار تشنجيانغ وييتشانغ وهانتشونغ كمواقع للدراسة. تتضمن مواقع الاختبار عيادتين للأمراض المنقولة بالاتصال الجنسي في المستشفيات ومؤسسة واحدة تعتمد على المجتمع MSM. سوف يولد المشاركون المؤهلون ذكرًا بيولوجيًا ، يبلغون من العمر 16 عامًا أو أكبر ، ويقومون بالإبلاغ عن ممارسة الجنس الشرجي السابق مع رجل آخر ، ولم يشاركوا أبدًا في برنامج الدفع المسبق ، بدون اختبار السيلان والكلاميديا السابق في الأشهر الـ 12 الماضية ، والمقيمين في الصين . بعد تصميم مجموعة عشوائية ، سيتم تخصيص كل مجموعة من عشرة مشاركين بشكل عشوائي في واحدة من ثلاثة أذرع: (1) ذراع الدفع إلى الأمام حيث يتم تقديم السيلان للرجال مجانا واختبار الكلاميديا ومن ثم سئل عما إذا كانوا يرغبون في التبرع ("دفعها للأمام") نحو اختبار اختبار المستقبل ؛ (2) ذراع دفع ما تريد ، حيث يتم تقديم اختبار مجاني للرجال ، ويُطلب منهم أن يقرروا المبلغ الذي يتعين عليهم دفعه بعد تلقي الاختبار ؛ (3) معيار ذراع الرعاية الذي يمكن للرجال أن يدفعوا الثمن الكامل لاختبار السيلان المزدوج والكلاميديا. والنتيجة الأولية هي اختبار السيلان / الكلاميديا المزدوج كما تم التحقق من السجلات الإدارية. تشمل النتائج الثانوية التكلفة الإضافية لكل اختبار ، التكلفة الإضافية لكل تشخيص ، الارتباط المجتمعي ، والتماسك الاجتماعي. سيتم احتساب النتيجة الأولية لكل ذراع باستخدام نية إلى المعالجة ومقارنتها باستخدام فواصل الثقة 95% من جانب واحد مع هامش زيادة بنسبة 20% على النحو المحدد التفوق.
مناقشة: سوف تدرس هذه الدراسة استراتيجية الدفع إلى الأمام مقارنةً بمعايير الرعاية في تحسين امتصاص اختبار السيلان والكلاميديا. سنستفيد من تجربة المجموعة العشوائية المضبوطة لتوفير أدلة علمية على التأثير المحتمل للدفع إلى الأمام. سوف تسلط نتائج هذه الدراسة الضوء على أساليب التدخل الجديدة لزيادة استخدام الخدمات الصحية الوقائية وابتكار طرق لتمويلها بين المجتمعات.

Translated from English version into Arabic by Ahmed Ibrahim, proofread by Amira Ali, through

TRANSLATORS
WITHOUT BORDERS

在中国男男性行为人群中通过接力检提高淋病和衣原体检测:三组随机对照试验的研究方案

张天舸，杨帆，唐卫明，Marcus Alexander，Laura Forastiere，Navin Kumar，Katherine Li，Fei Zou，杨立刚，米国栋，王晔华，黄文婷，Amy Lee，朱卫赞，Peter Vickerman，吴丹，杨斌，Nicholas A. Christakis，周海青

摘要

引言：淋病和衣原体检测在中国男男性行为者(MSM)中较差。一项类实验研究表明，一种“接力检”策略增加了 MSM 的淋病/衣原体双重检测。“接力检”向个人馈赠一份礼物(例如免费测试)，然后询问此人是否希望向他人赠送礼物。本研究报道了随机对照试验的研究方案，用于评估 MSM 在三组中对于淋病/衣原体双检的接受率和其他结果——一个“接力检”干预组、一只“任意付”干预组，和一个标准支付模作为对照组。

方法：将在广州和北京的 3 个艾滋病检测点招募 300 名 MSM。检测地点包括两个设在医院的 MSM 性传播疾病诊所和一个以 MSM 为基础的社区组织。符合条件的参与者为出生为男性，年龄为 16 岁或以上，报告曾与另一名男子肛交，从未参与“接力检“项目，在过去 12 个月内没有检测过淋病和衣原体，以及居住在中国。按照集群随机设计，每组 10 名参与者将被随机分配到三个组之一：(1) 在“接力检”组中，男性将免费获得淋病和衣原体检测，然后被询问他们是否愿意捐赠(将检测接力下去)，提供给未来来测试的人；(2) 在“任意付”组中，男性将免费获得检测，并被告知在接受测试后来决定支付多少费用；(3) 在标准支付组， 男性可以支付全款来获得淋病和衣原体双检。本研究主要结果是淋病/衣原体双检的检测率，经实验人员记录核实。次要结果包括每个测试的增量成本、每个诊断的增量成本、社区联系性和社会凝聚力。本研究将使用"治疗意向"计算每个组的主要结果，并使用单侧 95%置信区间进行比较，并将 20%的增幅定义为优势。

讨论：这项研究将探索与标准支付模式相比，“接力检”策略是否有效地提高淋病和衣原体的检测。本研究利用集群随机对照试验为“接力检”的潜在效应和优越性提供科学依据。这项研究的结果将揭示预防性保健服务利用的新干预方法，也将提供在社区中实施这种服务的可能卫生筹资方法。

实验注册: ClinicalTrials.gov，NCT03741725；注册于 2018 年 11 月 12 号。

Translated from English version into Chinese by Fan Yang

Pay-it-forward est un test de la gonorrhée et de la chlamydia chez les hommes ayant des rapports sexuels avec des hommes en Chine: protocole d'étude pour un essai contrôlé randomisé par grappes à trois groupes

Tiange P. Zhang, Fan Yang, Weiming Tang, Marcus Alexander, Laura Forastiere, Navin Kumar, Katherine Li, Fei Zou, Ligang Yang, Guodong Mi, Yehua Wang, Wenting Huang, Amy Lee, Weizan Zhu, Peter Vickerman, Dan Wu, Bin Yang, Nicholas A. Christakis, Joseph D. Tucker

Résumé

Contexte: Le dépistage de la gonorrhée et de la chlamydia est faible chez les hommes chinois ayant des rapports sexuels avec des hommes (HSH). Une étude quasi expérimentale a suggéré qu'une stratégie de pay-it-forward redoublerait le test de dépistage de la gonorrhée et de la chlamydia chez les HSH. Pay-it-forward offre un cadeau à l'individu (par exemple, un test gratuit), puis lui demande s'il souhaite faire un cadeau à une autre personne. Cet article présente le protocole d'un essai contrôlé randomisé visant à évaluer la double utilisation du test de dépistage de la gonorrhée / chlamydia et d'autres résultats chez les HSH en trois catégories: une à paiement anticipé, une à taux variable, et une standard de soins.

Méthodes: Trois cents HSH seront recrutés pour le test de dépistage du VIH en trois sites différents en Guangzhou et en Beijing. Les sites de test comprennent deux cliniques de traitement des maladies sexuellement transmissibles pour HSH en milieu hospitalier et un organisme communautaire pour HSH.

Les participants admissibles seront nés de sexe masculin, âgés de 16 ans ou plus, ayant déjà eu des relations sexuelles anales avec un autre homme, n'ayant jamais participé au programme Pay-it-forward, sans avoir subi de tests de dépistage de la gonorrhée et de la chlamydia au cours des 12 derniers mois, et résidant en Chine. Suivant un plan randomisé en grappes, chaque grappe de dix participants sera répartie au hasard dans l’une des trois catégories suivantes: (1) une catégorie pay-it-forward dans laquelle des hommes recevront des tests gratuits de dépistage de la gonorrhée et de la chlamydia et auxquels on demandera s’ils souhaitent faire un don (“Pay it forward”) vers les tests pour les futurs testeurs; (2) une catégorie payante dans laquelle on offre aux hommes un test gratuit et on leur demande de décider combien ils paieraient après s'être soumis au test; (3) une catégorie de soins standard dans laquelle les hommes peuvent payer le plein tarif du double test de dépistage de la gonorrhée et de la chlamydia. Le résultat principal est le double test de dépistage de la gonorrhée et de la chlamydia, vérifié par les dossiers administratifs. D'ultérieurs résultats sont le coût différentiel par test, le coût différentiel par diagnostic, la connectivité à la communauté et la cohésion sociale. Le résultat principal sera calculé pour chaque catégorie en utilisant l'intention de traiter et comparé en utilisant des intervalles de confiance unilatéraux à 95% avec une marge d'augmentation de 20% définie comme une supériorité.
Discussion: Cette étude examinera la stratégie du paiement pay-it-forward par rapport au traitement standard afin d'accroître l'adoption du test de la gonorrhée et de la chlamydia. Nous allons tirer profit de l’essai contrôlé randomisé par groupes pour fournir des preuves scientifiques de l’effet potentiel du paiement anticipé. Les résultats de cette étude feront la lumière sur les nouvelles méthodes d’intervention pour accroître l’utilisation des services de santé préventifs et sur les moyens novateurs de financement des communautés.

Translated from English version into French by Marina Della Torre, proofread by Imane Bouamoud, through

TRANSLATORS
WITHOUT BORDERS

Предоплаченное тестирование гонореи и хламидиоза у мужчин, имеющих секс с мужчинами, в Китае: протокол исследования для рандомизированного контролируемого исследования с участием трех групп

Тянге П. Чжан, Фан Ян, Веймин Тан, Маркус Александер, Лаура Форастьере, Навин Кумар, Кэтрин Ли, Фэй Цзоу, Лиганг Ян, Годун Ми, Йехуа Ван, Вентинг Хуан, Эми Ли, Вейцзан Чжу, Питер Викерман, Дан Ву, Бин Янг, Николас А. Кристакис, Джозеф Д.Такер

Аннотация

Предпосылки: Тестирование на гонорею и хламидиоз проводится редко среди китайских мужчин, имеющих секс с мужчинами (МСМ). Квазиэкспериментальное исследование показало, что стратегия «заплати вперед» увеличивает двойное тестирование гонореи/хламидиоза среди МСМ. "Заплати вперед" предлагает человеку подарок (например, бесплатный тест), а затем спрашивает того же человека, хочет ли он сделать подарок другому. В этой статье сообщается о протоколе рандомизированного контролируемого исследования, позволяющего оценить двойной

тест на гонорею/хламидиоз и другие результаты среди МСМ в трех группах - группа «заплати вперед», группа «заплати сколько хочешь» и группа стандартной медицинской помощи.

Методы: Триста МСМ будут выбраны на трех участках тестирования на ВИЧ в Гуанчжоу и Пекине. Участки тестирования включают в себя две больницы МСМ, специализирующиеся на заболеваниях, передаваемых половым путем, и одну организацию сообщества МСМ. Приемлемые участники должны быть биологически мужского пола в возрасте 16 лет и старше, которые сообщают о предыдущем анальном сексе с другим мужчиной, никогда не участвовавшими в программе "заплати вперед", без предварительного тестирования на гонорею и хламидиоз за последние 12 месяцев и проживать в Китае. Следуя кластерному рандомизированному исследованию, каждый кластер из десяти участников будет случайным образом распределен в одну из трех групп: (1) группа "заплати вперед", в которой мужчинам предлагается бесплатное тестирование на гонорею и хламидиоз, а затем спросят, хотят ли они пожертвовать ("заплати вперёд") для будущих участников; (2) группа «плати сколько хочешь», в которой мужчинам предлагается бесплатное тестирование и они сами решают, сколько платить после прохождения теста; (3) стандартное медицинское обслуживание, при котором мужчины могут заплатить полную цену за двойной тест на гонорею и хламидиоз. Первичным результатом является двойное тестирование на гонорею/хламидиоз, что подтверждается административными данными. Вторичные результаты включают в себя дополнительные затраты на тест, дополнительные затраты на постановку диагноза, связь с сообществом и социальную сплоченность. Первичный результат будет рассчитываться для каждой группы с использованием намерения лечить и сравниваться с использованием односторонних 95% доверительных интервалов с запасом 20%, определяемым как превосходство.

Обсуждение: В этом исследовании будет рассмотрена стратегия "заплати вперед" по сравнению со стандартом помощи в улучшении восприятия теста на гонорею и хламидиоз. Мы будем использовать кластерное рандомизированное контролируемое исследование для предоставления научных данных о потенциальном эффекте "заплати вперед". Результаты этого исследования позволят пролить свет на новые методы, направленные на повышение эффективности использования профилактических медицинских услуг, и определить новые способы их финансирования в сообществах.

Translated from English version into Russian by Anna Kukharchuk, proofread by Alexander Somin, through

TRANSLATORS
WITHOUT BORDERS

Pruebas de gonorrea y clamidia con cadena de favores entre hombres que han tenido relaciones sexuales con otros hombres en China: un protocolo de estudio para un ensayo aleatorio controlado de tres ramas.

Tiange P. Zhang, Fan Yang, Weiming Tang, Marcus Alexander, Laura Forastiere, Navin Kumar, Katherine Li, Fei Zou, Ligang Yang, Guodong Mi, Yehua Wang, Wenting Huang, Amy Lee, Weizan Zhu, Peter Vickerman, Dan Wu, Bin Yang, Nicholas A. Christakis, Joseph D. Tucker

Resumen

Introducción: las prubas de gonrrea y clamidia son deficientes entre hombres chinos que han tenido relaciones sexuales con otros hombres (MSM, por sus siglas en inglés). Un estudio cuasiexperimental sugirió que una estrategia de cadena de favores incrementó las pruebas dobles de gonorrea y clamidia entre MSM. La cadena de favores ofrece un obsequio individual (p. ej. una prueba gratuita) y se pide después a la misma persona si le gustaría darle un obsequio a otro individuo. Este artículo informa sobre el protocolo de un ensayo aleatorio controlado para evaluar el uso de las pruebas dobles de gonorrea y clamidia y otros resultados entre MSM en tres ramas: uno de cadena de favores, otro de "paga lo que quieras" y otro de atención médica estándar.

Métodos: Se reclutarán trescientos MSM en tres centros de pruebas de VIH en Cantón y Pekín. Los centros de pruebas incluyen clínicas para MSM con enfermedades de transmisión sexual basadas en dos hospitales y una organización comunitaria de MSM. Los participantes aptos habrán sido nacidos hombres biológicamente, de 16 años en adelante, que hayan tenido sexo anal previamente con otro hombre, que nunca hayan participado en un programa de cadena de favores, sin pruebas previas de gonorrea y clamidia en los pasados 12 meses y residentes en China. Siguiendo un diseño grupal aleatorizado, se asignará cada grupo de diez participantes de forma aleatoria a una de los tres ramas: (1) una de cadena de favores en el que se ofrece a los hombres pruebas gratuitas de gonorrea y clamidia y se les pregunta si les gustaría donar ("pasar el favor") para las pruebas de futuros sujetos; (2) otro de "paga lo que quieras" en el que también se les ofrecen estas pruebas gratuitamente y ellos mismos deciden cuánto desean pagar por recibirlas; y (3) otro de atención médica estándar en el que pueden pagar el precio completo de estas pruebas dobles. El resultado principal es la verificación de las pruebas dobles de gonorrea y clamidia en los registros administrativos. Los resultados secundarios incluyen los costes adicionales por prueba y por diagnóstico, el vínculo entre la comunidad y la cohesión social. El resultado principal se calculará para cada rama según la intención de tratamiento y se contrastará mediante intervalos de confianza unilaterales del 95% con un margen de incremento del 20% definido como superioridad.

Discusión: este estudio examinará la estrategia de las cadenas de favores en comparación con la atención médica estándar a la hora de mejorar la aceptación de las pruebas de gonorrea y clamidia. Aprovecharemos el ensayo aleatorio controlado grupal para proporcionar pruebas científicas sobre el efecto potencial de las cadenas de favores. Las conclusiones de este estudio arrojarán luz sobre los métodos de intervención innovadores para aumentar la utilización de los servicios de salud preventivos e introducir nuevas formas de financiarlos en las comunidades.

Translated from English version into Spanish by Carolina Forte, proofread by Laura Woolley-Nunez, through

TRANSLATORS WITHOUT BORDERS
