# Supplementary material for: Pay-it-forward gonorrhea and chlamydia testing among men who have sex with men in China: a study protocol for a three-arm cluster randomized controlled trial
Source: Infect Dis Poverty. 2019 Aug 16;8:76. doi: 10.1186/s40249-019-0581-1 (PMC6700988; doi:10.1186/s40249-019-0581-1)
Supplement: Supplementary file 2 — Survey instrument (English version). This is the English version of the survey for our study. (DOCX 74 kb) [file 40249_2019_581_MOESM2_ESM.docx]

**A. Sociodemographics**

*The next set of questions will ask you to provide some information about yourself.*

A1. Age: ____ years old

A2. Nationality

1. Han Chinese 2. Other _________

A3. Current marital status:

1. Never married
2. Engaged or Married
3. Separated or divorced
4. Widowed

A4. Highest level of completed education:

1. Elementary
2. Middle school
3. High school or vocational school
4. Bachelor or associate degree
5. Above bachelor’s degree

A5. What is your occupation?

o Student

o Civil servant

o Farmer

o Labor worker (blue collar)

o Office worker (white collar)

o Seller/service staff

o Technician

o Unemployed

o Other______

A6. What is your total individual **monthly** income from all sources?

1. <1500 RMB/month
2. 1500-3000 RMB/month
3. 3001-5000 RMB/month
4. 5001-8000 RMB/month
5. >8000 RMB/month

A7. What is your gender identity?

1. Male
2. Female
3. Transgender
4. Unsure/Other

A8. What is your sexual orientation?

1. Homosexual
2. Bisexual
3. Heterosexual
4. Unsure/Other

**B. Sexual behaviors**

*The next set of questions will ask you about your sexual behaviors with other men.*

B1. What is your role during anal sex?

1. Mostly receptive (bottom)
2. Mostly insertive (top)
3. Half and half (versatile)

B2. In the past 3 months, how many sex partners have you had? (Number)

____ partners

B3. In the past 3 months, have you had anal sex?

1. Yes
2. No (Skip to B5)

B4. In the past 3 months, when you had anal sex, how frequently did you use condoms?

1. 0% condom use
2. Less than 50% condom use
3. More than 50% condom use
4. 100% condom use

B5. In the past 3 months, have you had condomless vaginal sex?

1. Yes
2. No

B6. In the past 3 months, have you had condomless oral sex?

1. Yes
2. No

B7. In the past, have you told anyone about your sexuality or sexual history with men? (Select all that apply)

1. Yes, my long-term female partner/wife
2. Yes, my family members
3. Yes, my friends
4. Yes, my healthcare providers
5. Yes, others:_________
6. No one

**C. Clinical Information**

C1. Do you have any symptoms that you are worried may be due to an STI?

1. Yes. Symptoms: _____________
2. No

C2. Have you ever tested for HIV in the past?

1. Yes
2. No (Skip to C5)

C3. When was the last time you tested for HIV? (If cannot recall exactly, please estimate)

Year:________Month:______Day:________

C4. In the last two years, how frequently did you get tested for HIV?

1. Less than once every two years
2. Once a year
3. Once every six months
4. Once every three months
5. Monthly

C5. Today, did you agree to get tested for gonorrhea and chlamydia?

1. Yes (Go to C6)
2. No (Go to C7)

C6 (Pay-it-forward arm). If you agreed to testing for gonorrhea and chlamydia today, what is the MAIN reason? (**Choose ONE**)

1. “Pay It Forward” allowed for discounted testing
2. “Pay It Forward” allowed paying kindness forward to community members
3. Recent symptoms
4. Recent high-risk sexual behavior
5. Testing site’s staff told me to get tested
6. A friend told me to get tested
7. Other ____________________

C6 (Pay-what-you-want arm). If you agreed to testing for gonorrhea and chlamydia today, what is the MAIN reason? (**Choose ONE**)

1. “Pay What You Want” allowed for discounted testing
2. Recent symptoms
3. Recent high-risk sexual behavior
4. Testing site’s staff told me to get tested
5. A friend told me to get tested

Other ____________________

C6 (Standard of care arm). If you agreed to testing for gonorrhea and chlamydia today, what is the MAIN reason? (**Choose ONE**)

1. Because the research staff introduced gonorrhea and chlamydia testing
2. Recent symptoms
3. Recent high-risk sexual behavior
4. Testing site’s staff told me to get tested
5. A friend told me to get tested
6. Other ____________________

C7. If you did NOT agree to testing for gonorrhea and chlamydia today, why NOT? (select all that apply)

1. I don’t know anything about gonorrhea or chlamydia
2. I don’t want to know if I have gonorrhea or chlamydia
3. I don’t need to get tested
4. Too much of a hassle
5. Too expensive
6. I am worried about confidentiality
7. I am afraid of pain/ discomfort
8. I am embarrassed to get a sample taken
9. I am embarrassed to get tested in front of my friend/partner
10. I am afraid that my results will be positive
11. Other ____________________

**D. Community Engagement**

*The next set of questions asks about your experiences with MSM-related causes, events and organizations in your community.*

D1. Have you ever participated in online forums or discussions on social media (ie. Weixin, Weibo, Twitter, or other on-line communities) about issues related to the MSM community?

o Yes

o No

D2. Are you aware of any ongoing MSM-related community events?

o Yes

o No

D3. Have you ever encouraged someone to use MSM-related community resources, such as free HIV and syphilis testing services?

o Yes

o No

D4. Have you ever attended MSM-related community events?

o Yes

o No

D5. Have you ever donated to MSM-related causes, events, or organizations? (other than today)

o Yes

o No

D6. Have you ever volunteered for MSM-related causes, events, or organizations?

o Yes

o No

**E. Community Connectedness**

*The following set of questions asks about your feelings toward the MSM. Here, “MSM community” broadly refers to the collective of individuals and community organizations that have an interest in MSM-related issues.*

E1. You feel that you are a part of the MSM community.

-
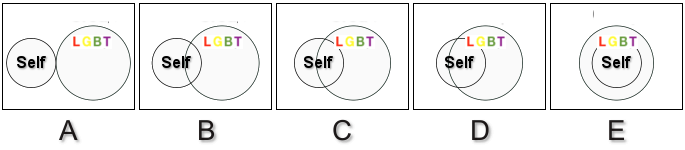
Strongly Agree
- Agree
- Disagree
- Strongly Disagree

E2. Participating in the MSM community is a positive thing for you.

- Strongly Agree
- Agree
- Disagree
- Strongly Disagree

E3. You are proud of the MSM community.

- Strongly Agree
- Agree
- Disagree
- Strongly Disagree

E4. It is important for you to be an advocate for the MSM community.

- Strongly Agree
- Agree
- Disagree
- Strongly Disagree

E5. If you and your peers work together, the problems in the MSM community can be solved.

- Strongly Agree
- Agree
- Disagree
- Strongly Disagree

E6. You really feel that any problems faced by the MSM community are also your own problems.

- Strongly Agree
- Agree
- Disagree
- Strongly Disagree

E7. The diagram below is designed to represent your relationship (“Self”) with LGBT as a group ("LGBT"). Please indicate your relationship by selecting the option that best captures your relationship with this LGBT as a group.

**F. Social Cohesion**

F1. You can count on other MSM in your group of friends if you need to borrow money.

- Strongly Agree
- Agree
- Disagree
- Strongly Disagree

F2. You can count on other MSM in your group of friends if you need to talk about your problems.

- Strongly Agree
- Agree
- Disagree
- Strongly Disagree

F3. You can count on other MSM in your group of friends if you need somewhere to stay.

- Strongly Agree
- Agree
- Disagree
- Strongly Disagree

F4. The group of MSM with whom you socialize with is an integrated group.

- Strongly Agree
- Agree
- Disagree
- Strongly Disagree

F5. You can trust the majority of the MSM you know.

- Strongly Agree
- Agree
- Disagree
- Strongly Disagree

F6. In general, MSM in your group of friends in the area where you live only worry about themselves

- Strongly Agree
- Agree
- Disagree
- Strongly Disagree

F7. In general the MSM you socialize with are always arguing amongst each other

- Strongly Agree
- Agree
- Disagree
- Strongly Disagree

**G. Pay-It-Forward Participation (Pay-it-forward arm only)**

G1. Today, you came to testing:

1. By yourself (Skip to G3)
2. Accompanied by someone else

G2. How would you describe your relationship to the person accompanying you?

1. Sex partner

2. MSM peer

3. Non-MSM peer

4. Family

5. Other, specify:____

G3. Did you choose to contribute any amount of money?

1. Yes
2. No

G4. What determined your contribution amount?

1. One’s own financial situation

2. Normal price of testing

3. Quality of testing service

4. Estimate of how much others contributed

5. Feel bad if not pay anything

6. Other, specify:______

G5. What do you believe are the main benefits to participating in the PIF program? (select all that apply)

1. I can receive discounted GC/CT test
2. I can experience warm glow through receiving donated testing
3. It reduces my STI risk by making my community healthier
4. It can help more MSM get tested
5. It allows someone to help me, and then I can help someone else
6. Other ____________________

**G. Pay-What-You-Want Participation**

**(Pay-what-you-want arm only)**

G1. Today, you came to testing:

1. By yourself (Skip to G3)
2. Accompanied by someone else

G2. How would you describe your relationship to the person accompanying you?

1. Sex partner

2. MSM peer

3. Non-MSM peer

4. Family

5. Other:____

G3. Did you choose to contribute any amount of money?

1. Yes
2. No

G4. What determined your contribution amount?

1. One’s own financial situation

2. Normal price of testing

3. Quality of testing service

4. Estimate of how much others contributed

5. Feel bad if not pay anything

6. Other:_______

G5. What do you believe are the main benefits to participating in the PIF program? (select all that apply)

1. I can receive discounted GC/CT test
2. I can experience warm glow through receiving discounted testing
3. It reduces my STI risk by making my community healthier
4. It can help more MSM get tested
5. It allows MSM to have more control over testing price
6. Other ____________________
