# Supplementary material for: Pay-it-forward gonorrhea and chlamydia testing among men who have sex with men in China: a study protocol for a three-arm cluster randomized controlled trial
Source: Infect Dis Poverty. 2019 Aug 16;8:76. doi: 10.1186/s40249-019-0581-1 (PMC6700988; doi:10.1186/s40249-019-0581-1)
Supplement: Supplementary file 3 — Introduction to Gonorrhea and Chlamydia pamphlet (English version). This is the English version of the pamphlet used by study organizers to introduce Gonorrhea and Chlamydia to eligible men. (PPTX 779 kb) [file 40249_2019_581_MOESM3_ESM.pptx]

## Slide 1
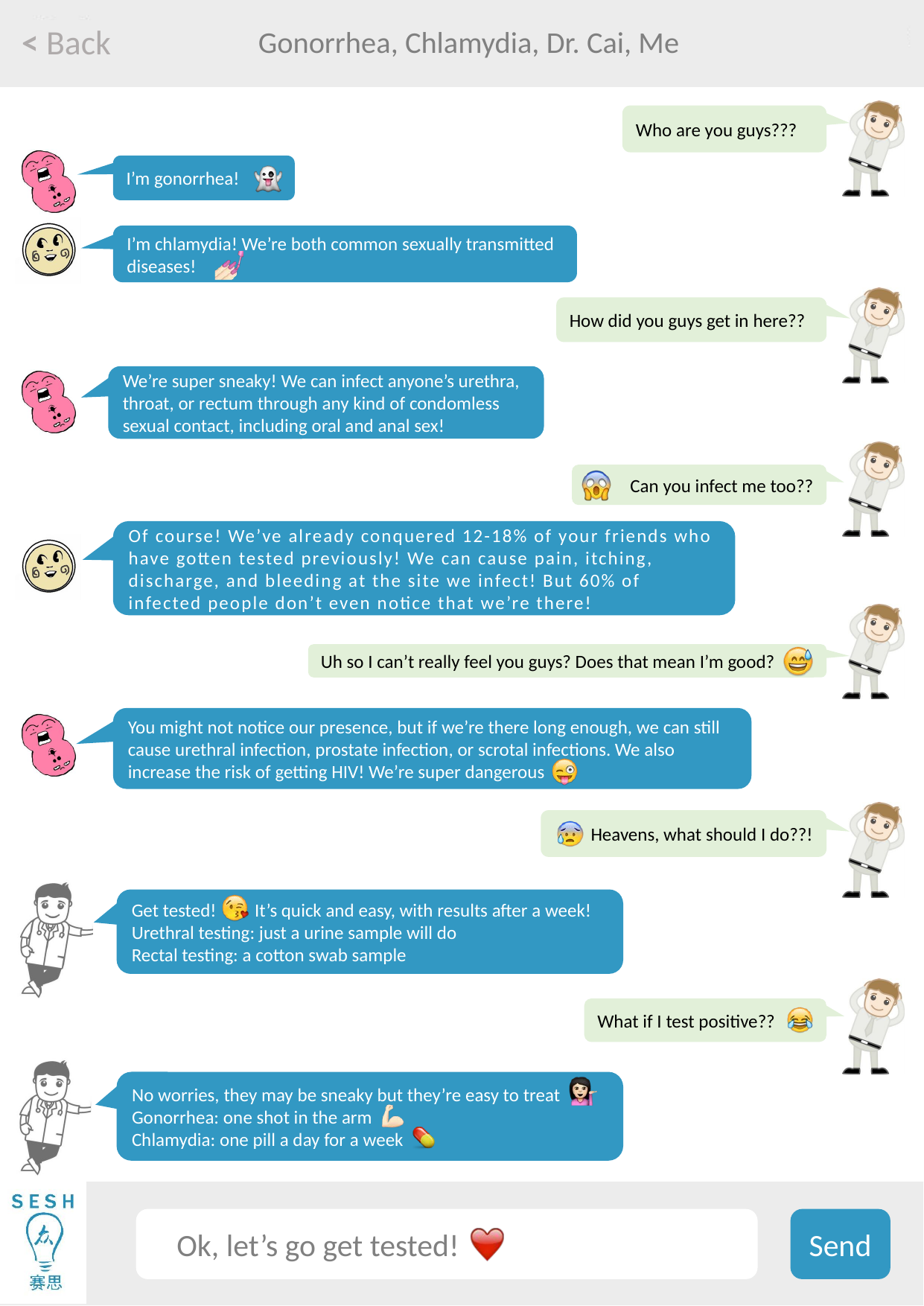

Gonorrhea, Chlamydia, Dr. Cai, Me
< Back
Who are you guys???
I’m gonorrhea!
I’m chlamydia! We’re both common sexually transmitted diseases!
How did you guys get in here??
We’re super sneaky! We can infect anyone’s urethra, throat, or rectum through any kind of condomless sexual contact, including oral and anal sex!
Can you infect me too??
Of course! We’ve already conquered 12-18% of your friends who have gotten tested previously! We can cause pain, itching, discharge, and bleeding at the site we infect! But 60% of infected people don’t even notice that we’re there!
Uh so I can’t really feel you guys? Does that mean I’m good?
You might not notice our presence, but if we’re there long enough, we can still cause urethral infection, prostate infection, or scrotal infections. We also increase the risk of getting HIV! We’re super dangerous
Heavens, what should I do??!
Get tested! It’s quick and easy, with results after a week!
Urethral testing: just a urine sample will do
Rectal testing: a cotton swab sample
What if I test positive??
No worries, they may be sneaky but they’re easy to treat
Gonorrhea: one shot in the arm
Chlamydia: one pill a day for a week
Send
Ok, let’s go get tested!
