# Supplementary material for: Pay-it-forward gonorrhea and chlamydia testing among men who have sex with men in China: a study protocol for a three-arm cluster randomized controlled trial
Source: Infect Dis Poverty. 2019 Aug 16;8:76. doi: 10.1186/s40249-019-0581-1 (PMC6700988; doi:10.1186/s40249-019-0581-1)
Supplement: Supplementary file 4 — Introduction to Pay-It-Forward pamphlet (English version). This is the English version of the pamphlet used by study organizers to introduce pay-it-forward to eligible men. (PPTX 311 kb) [file 40249_2019_581_MOESM4_ESM.pptx]

## Slide 1
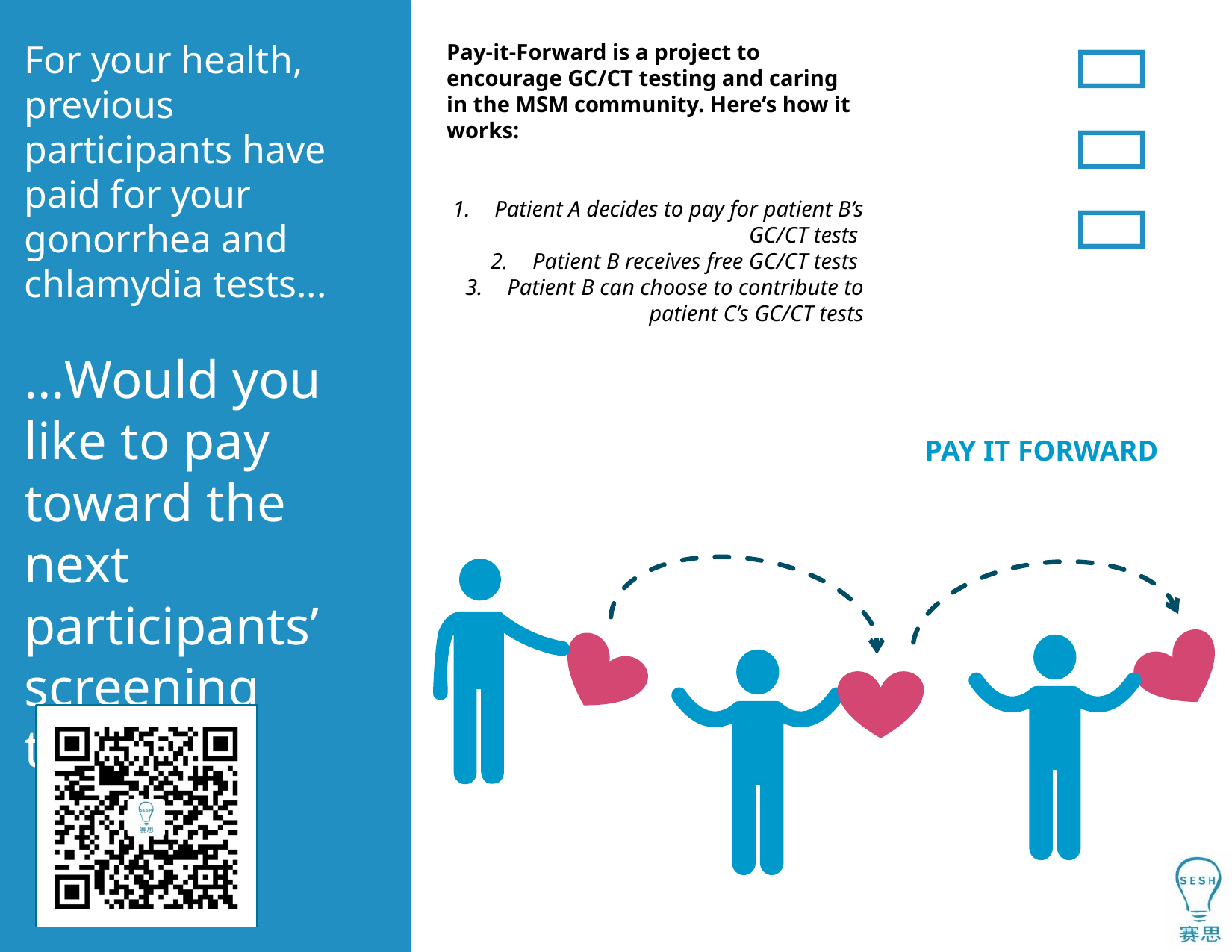

For your health, previous participants have paid for your gonorrhea and chlamydia tests...
接 力 检
Pay-it-Forward is a project to encourage GC/CT testing and caring in the MSM community. Here’s how it works:
Patient A decides to pay for patient B’s GC/CT tests
Patient B receives free GC/CT tests
Patient B can choose to contribute to patient C’s GC/CT tests
…Would you like to pay toward the next participants’ screening tests?
PAY IT FORWARD
支付二维码GOES HERE
