# Supplementary material for: Pay-it-forward gonorrhea and chlamydia testing among men who have sex with men in China: a study protocol for a three-arm cluster randomized controlled trial
Source: Infect Dis Poverty. 2019 Aug 16;8:76. doi: 10.1186/s40249-019-0581-1 (PMC6700988; doi:10.1186/s40249-019-0581-1)
Supplement: Supplementary file 5 — Informed consent form (English version). This is the English version of the informed consent form for our study. (DOCX 47 kb) [file 40249_2019_581_MOESM5_ESM.docx]

**Informed Consent Form**

**Title of Study: Pay-it-forward gonorrhea and chlamydia testing among men who have sex with men in China: a cluster randomized controlled trial**

**IRB study number:** 18-2142 **Principal Investigator:** Dr. Joseph D. Tucker
Dr. Joseph D. Tucker, UNC Project-China, Number 2 Lujing Road, Guangzhou, China,

**What are some general things you should know about research studies?** You are being asked to participate in a research study. To join this research study is voluntary. You may for whatever reason refuse to join or withdraw your consent to be in the study at any time, without penalty. Details about this study are discussed below. It is important that you understand this information so that you can make an informed choice about joining this research study.

**What is the purpose of this study?** Innovative approaches to promote **gonorrhea/chlamydia testing** are urgently needed. One pilot study in Guangzhou found that a pay-it-forward model increased gonorrhea/chlamydia testing 19-fold. Pay-it-forward gives a gift to one person, then asks the same person if they would like to give a gift to another person. The effectiveness and mechanisms of pay-it-forward models remain poorly understood. This randomized controlled trial will evaluate dual gonorrhea/chlamydia test uptake and other outcomes among MSM in three trial arms: (1) a pay-it-forward arm in which men are given a gift of free testing and asked about whether they would like to donate to future testers, (2) a pay-what-you-want arm in which men are offered gonorrhoea and chlamydia testing and told that they decide how much to pay after receiving the test, (3) a standard of care arm in which men can pay the full price for dual gonorrhoea/chlamydia testing.

**How many people will take part in this study?** If you decide to participate in this research study, you will be one of approximately 300 individuals recruited across Guangzhou.

**What will happen if you take part in the study?** Your part in this study will last approximately 30 minutes. During this, you will be offered dual gonorrhea/chlamydia testing and be asked to complete an online questionnaire. The study questionnaires will ask you to provide information on socio-demographics, sexual behaviors, HIV/STI testing history, psychosocial conditions, and social networks. You will then be assigned to one of the three groups and asked if you want to be tested for gonorrhea and chlamydia. You will not be able to decide which group you are assigned to. The three groups will have different models for delivering gonorrhea and chlamydia testing. You will then receive testing through the clinic. The clinic staff will contact you with the results, and refer you for treatment. If you test positive and need treatment for the infection, the clinic staff will help you get treatment. This is not part of the study and will not be paid for.

**What are the possible benefits from being in this study?** Research is designed to benefit society by gaining new knowledge. The proposed study will make important contributions to the sexual health literature. The field of gonorrhea/chlamydia testing interventions among young MSM in resource-limited settings is in its infancy. The results from this study will help the research team develop a MSM targeted, community-level intervention that will be fielded and evaluated in the Chinese setting. Your participation will also help design better interventions to promote gonorrhea/chlamydia testing among MSM in China.

**What are the possible risks or discomforts involved from being in this study?** We will ask participants to provide sensitive information about their sexual partners and practices. Participants may feel embarrassed, anxious, or otherwise distressed by providing information of such a personal nature. Participants may also experience fatigue in response to the proposed evaluations (e.g. from looking at a smartphone screen). Some participants might fear that refusal to participate in the study might jeopardize their sexual orientation identity – especially if the participant has not come “out” to him or herself and/or the community). Other participants may fear that the research staff might “out” them or discuss their private details with other (MSM and non-MSM) members in their community. While the risk is minimal, there is still the possibility for breaches of confidentiality.

**How will your privacy be protected?** All data are directly entered into computers as participants complete the questionnaires. Programs to ensure accuracy, completeness, and internal consistency are automated. Data can be readily downloaded and converted to the format of commercially available statistical software. During collection of the online portion of the study, all data will be transmitted securely using SSL (TLS) 128 bit encryption across the Internet (HTTP). SSL providers users with the assurance of access to a valid, “non-spoofed” site, and prevents data interception or tampering with sensitive information. The SSL certificate that will be used for this project will use 128-bit encryption, the preferred security level of government and financial institutions. 128-bit encryption offers protection that is virtually unbreakable. For example, if a hacker could crack a standard 40-bit SSL session in a day, it is estimated that it would take well beyond a trillion years to accomplish the same thing against a 128-bit SSL session. A dedicated server, which eliminates security issues involved with shared hosting environments where hundreds of websites and users reside on one shared web server as well as ensuring both physical and network security, will be used to house the data. Data will be located in a secured server at UNC Chapel Hill.

The server will be configured with redundant hard drive array to ensure reliability. Access to the data will be password protected within the server’s firewall. Survey responses will be kept separately from participants’ email addresses; the two files will be linked with a non-descript, unique, randomly generally identifier. Only the PI and a designated senior staff member will have the password to access to the “key” that links the nondescript identifier to personally identifiable information. Cookies will not be used in any way to track participant activity.

**What if you want to stop before your part in the study is complete? If at any point in the study you do not want to answer a question or no longer want to participate,** you can stop and withdraw from this study without penalty. The investigators also have the right to stop your participation if you have an unexpected reaction, have failed to follow instructions, etc.

**Will you receive anything for being in this study? Will it cost anything?**

**Participants will receive a small stationery gift valued at approximately 1 USD upon completion of the survey. The cost of the gonorrhea/chlamydia test offered will depend on which group you are randomized into, but you will not have to pay more than your usual test fee. If you decide to take the test and the results are positive, the cost of treatment will not be paid for by the study.**

**What if you have questions about this study?** If you have any questions, complaints, or concerns about the research or your participation in the study, feel free to contact Tiange Zhang (18611636966).

**What if you have questions about your rights as a research participant?** All research on human volunteers is reviewed by a committee that works to protect your rights and welfare. If you have questions or concerns, or if you would like to obtain information or offer input, please contact the Institutional Review Board at 020-87255824 or by email to [IRB_subjects@unc.edu](mailto:IRB_subjects@unc.edu). You may also contact the Guangdong Provincial Skin Diseases & STI Control Center IRB at 020－83027652 or by email to [sesh@seshglobal.org](mailto:sesh@seshglobal.org).

If you understand and agree to participate in this research study, please select “Agree” from the options below. We thank you for your participation!

- Agree
- Decline (Skip to End of Survey)

**Signature/Date_____________________**
